# LC-ESI-MS/MS reveals the formation of reactive intermediates in brigatinib metabolism: elucidation of bioactivation pathways

**DOI:** 10.1039/c7ra10533a

**Published:** 2018-01-03

**Authors:** Adnan A. Kadi, Mohamed W. Attwa, Hany W. Darwish

**Affiliations:** Department of Pharmaceutical Chemistry, College of Pharmacy, King Saud University P. O. Box 2457 Riyadh 11451 Kingdom of Saudi Arabia mzeidan@ksu.edu.sa +966 1146 76 220 +966 1146 70237; Analytical Chemistry Department, Faculty of Pharmacy, Cairo University Kasr El-Aini St. Cairo 11562 Egypt

## Abstract

Brigatinib (BGB) is a newly approved anaplastic lymphoma kinase (ALK) inhibitor. On April 28, 2017, BGB was approved by the U.S. FDA for the treatment of metastatic anaplastic lymphoma kinase-positive non-small cell lung cancer. The toxicity profile of BGB includes nausea, fatigue, diarrhea, elevated lipase, dyspnoea, hypertension, hypoxia, pneumonia, elevated amylase, pulmonary embolism, elevated ALT, hyponatraemia and hypophosphatemia. Using LC-MS/MS, we investigated the *in vitro* phase I metabolism of for BGB in rat liver microsomes (RLMs). In the *in vitro* metabolism of BGB, iminium reactive intermediates were trapped by potassium cyanide forming a stable complex that can be characterized by LC-MS/MS. Four BGB *in vitro* phase I metabolites were identified. *In vitro* phase I metabolic pathways were *N*-dealkylation, α hydroxylation and α oxidation. Additionally, three iminium reactive metabolites were found and the bioactivation mechanisms were proposed. A piperidine ring was found to be responsible for BGB bioactivation. The presence of these three reactive metabolites may be the main reason for BGB side effects. A literature review showed no previous article reported the *in vitro* phase I metabolism study of BGB or structural identification of the formed reactive metabolites.

## Abstract 2

Four phase I BGB metabolites and three cyano adducts for BGB were detected using LC-MS/MS. The piperidine ring was found to be responsible for BGB bioactivation and the bioactivation pathways are proposed.

## Graphical abstract

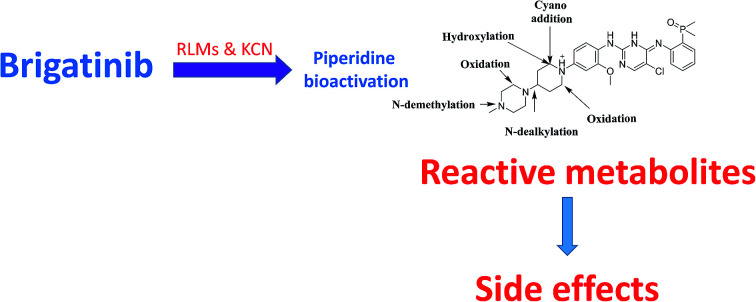

## 1. Introduction

Lung cancer is considered the most common reason of death from cancer worldwide. It is considered to be responsible for almost 20% of total cancer deaths (1.59 million deaths) in 2012.^1^ Non-small cell lung cancers (NSCLCs) represented about 90% of lung cancers, which include a number of subtypes driven by various activated oncogenes.^2,3^ New advances in molecular profiling technologies have considerably enhanced the development of personalised therapies based on individual genetic or protein profiles.^4,5^ Targeted personalised therapies achieved great success in treating NSCLC patients.^6–8^ ALK is an insulin receptor tyrosine kinase family (RTK) member.^9^ ALK inhibitors are anti cancer drugs that act on tumours with variations of ALK such as an EML4-ALK translocation.^10^ EML4-ALK translocations represented about 4–7% of non-small cell lung carcinomas (NSCLC).^11^

BGB (ALUNBRIG tablets) is an orally available ALK inhibitor that demonstrated ability to overcome crizotinib resistance mutations.^12,13^ On April 28, 2017, BGB was approved by the US FDA for the treatment of patients with metastatic ALK-positive NSCLC that have progressed on or are intolerant to crizotinib.

Metabolism is considered a detoxification process that responsible for increasing of the hydrophilicity of xenobiotics and endogenous compounds to be easily excreted from the body. Metabolites are usually less toxic if compared to parent molecules, but in rare cases bioactivation occurred forming reactive intermediates that can covalently modify proteins initiating drug-organ toxicities steps.^14–18^ Reactive intermediates are generated in most cases from phase I metabolism and are considered crucial in drug induced toxicity. Reactive metabolites such as iminium ion are unstable, formed in small amounts, and react rapidly with nucleophiles within matrix such as proteins and can, therefore, not found in regular metabolism studies. Hard electrophiles (such as iminium ions) react with hard nucleophiles, such as lysine residues in proteins and basic groups in DNA.^19^ Methods used to trap reactive intermediates are well established and were used for screening of these intermediates in early drug discovery.^20^ Direct detection and characterization of reactive intermediates are not possible due to their unstable and transient nature, so trapping agent was used forming adducts that are stable and can be analyzed by tandem mass spectrometry.^21,22^

The low concentration (nM) of metabolites and the complex nature of biological matrices made metabolite characterization an analytical challenge. Liquid chromatography mass spectrometry (LC-MS) has become the analytical tool of choice for the detection and identification of metabolite as it is characterized by high sensitivity, selectivity and its ability to separate, detect, and identify many metabolites in the presence of complex matrix.^23^

**Fig. 1:**
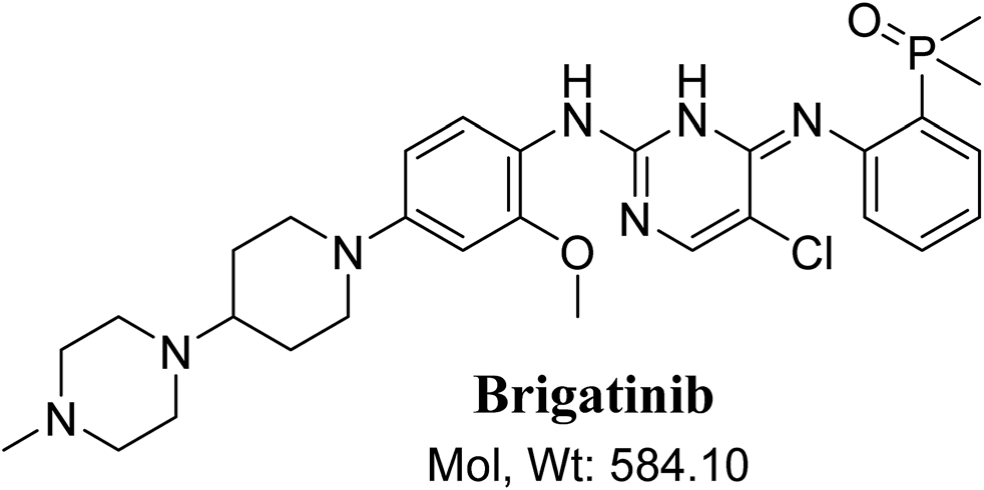
Chemical structure of brigatinib.

BGB chemical structure contains *N*-methyl piperazine and piperidine groups which are cyclic tertiary amine groups (Fig. 1) that undergo bioactivation forming iminium ion intermediates^24–26^ that are hard electrophiles and can be trapped efficiently by KCN.^14,24,25,27^ The formed adducts can be separated, detected and characterized using LC-MS/MS.^21,22,24,28,29^

In our work, we are profiling unreported *in vitro* phase I metabolites of brigatinib and we are trying to give insight about the reasons which may be responsible for the observed side effects of BGB. The toxicity profile of BGB includes nausea, fatigue, diarrhea, elevated lipase, dyspnoea, hypertension, hypoxia, pneumonia, elevated amylase, fatigue, pulmonary embolism, elevated ALT, hyponatraemia and hypophosphatemia.^30^ We are illuminating the way towards further developing ALK inhibitors with lower side effects. *N*-Demethylated *in vitro* phase I metabolite of BGB was reported,^31^ while other metabolites or adducts were not reported. Although BGB contains *N*-methyl piperazine group, we found that it had no role in bioactivation of BGB that is differ than other TKIs.^27^ Piperidine group of BGB was found to be responsible for BGB bioactivation.

## 2. Chemicals and methods

### 2.1. Chemicals

BGB was procured from Med Chem. Express (Princeton, NJ, USA). HPLC-grade Acetonitrile (ACN), potassium cyanide (KCN) and formic acid were purchased from Sigma-Aldrich (West Chester, PA, USA). Purified water was obtained from in-house Milli-Q plus purification system. RLMs were prepared in-house using Sprague Dawley rats following a previously published method.^32^ Sprague Dawley rats were obtained from Animal Care Center, College of Pharmacy, King Saud University. Animals' were maintained following the guidelines of Animal Care Center, College of Pharmacy, King Saud University and approved by Local Animal Care and Use Committee of King Saud University.

### 2.2. LC-MS/MS chromatographic parameters

LC-MS/MS parameters are summarized in Table 1. Slow gradient binary mobile phase is used to separate closely related metabolites. Positive mode was used for BGB detection as BGB chemical structure contains many basic nitrogen that can be easily protonated and this matched with the literature.^33^ Product ions (PIs) of BGB, *in vitro* phase I metabolites and cyano adducts in the collision cell. LC-MS/MS system and data acquisition were controlled by Mass Hunter software.

**Table 1:** LC-MS/MS method parameters

Liquid chromatographic parameters			Mass spectrometric parameters	
HPLC	Agilent 1200		Mass spectrometer	Agilent 6410 QQQ
Gradient binary mobile phase	A: 0.1% formic acid in H_2_O		Ionization source	Drying gas: N_2_ gas
	B: ACN			Flow rate (12 L min^−1^)
	Flow rate: 0.3 mL min^−1^			Pressure (60 psi)
Agilent eclipse plus C_18_ Column	150 mm length			Desolvation temperature: 350 °C
	2.1 mm internal diameter			Capillary voltage: 4000 V
	3.5 μm particle size		Collision gas	High purity N_2_
	Temperature: 22 ± 1 °C			
Gradient system	Time (min)	% B	Mass parameters	Mode: positive product ion (PI)
	(0–5)	5%		Fragmentor voltage: 145 V
	(5–60)	5 to 40%		
	(60–80)	40 to 90%		Capillary voltage: 4000 V
	(80–85)	90 to 5%		
Run time	85 min			Collision energy: 30 eV
Post time	15 min			
Injection volume	15 μL			
Analytes	Brigatinib, *in vitro* phase I metabolites and cyano adducts			

### 2.3. RLMs incubations of BGB

Ten μM BGB was incubated with 40 μL RLMs (1.0 mg mL^−1^), NADPH (1.0 mM) and Na/K phosphate buffer (50 mM containing 3.3 mM MgCl_2_, pH 7.4). Metabolic mixtures were kept for 2 hours in a shaking water bath at 37 °C and then the reactions were stopped by adding 2 mL of ice-cold ACN for protein precipitation. Centrifugation was done at 14 000 rpm, 10 min and 4 °C to devoid of precipitated proteins. The supernatants were removed, evaporated and reconstituted in the mobile phase at the start of the gradient system (5% ACN/95% (0.1% formic acid in water)). The same experiment of RLMs incubation was repeated with 1.0 mM KCN to capture bioactive metabolites. Incubations were performed in triplicate to confirm the obtained results.

### 2.4. Identification of phase I and reactive metabolites of BGB

Full MS scan and PI modes were used for identification and characterization of BGB, its *in vitro* phase I metabolites and cyano adducts. In order to get as much qualitative information as we can, we used PI mode and we used all fragments. PI mode gave more qualitative information comparing to multiple reaction monitoring mode which is mainly used for quantification as stated in the LC-MS/MS method for quantification of BGB.^33^ Extracted ion chromatogram (EIC) of *m*/*z* of the supposed BGB related metabolites was used to locate and characterize *in vitro* phase I and reactive metabolites in the total ion chromatogram of the metabolic mixture extract. Comparison of the EICs of the metabolic extract was done with control incubations (that does not contain either BGB or RLMs).

## 3. Results and discussion

### 3.1. PI study of BGB

**Fig. 2:**
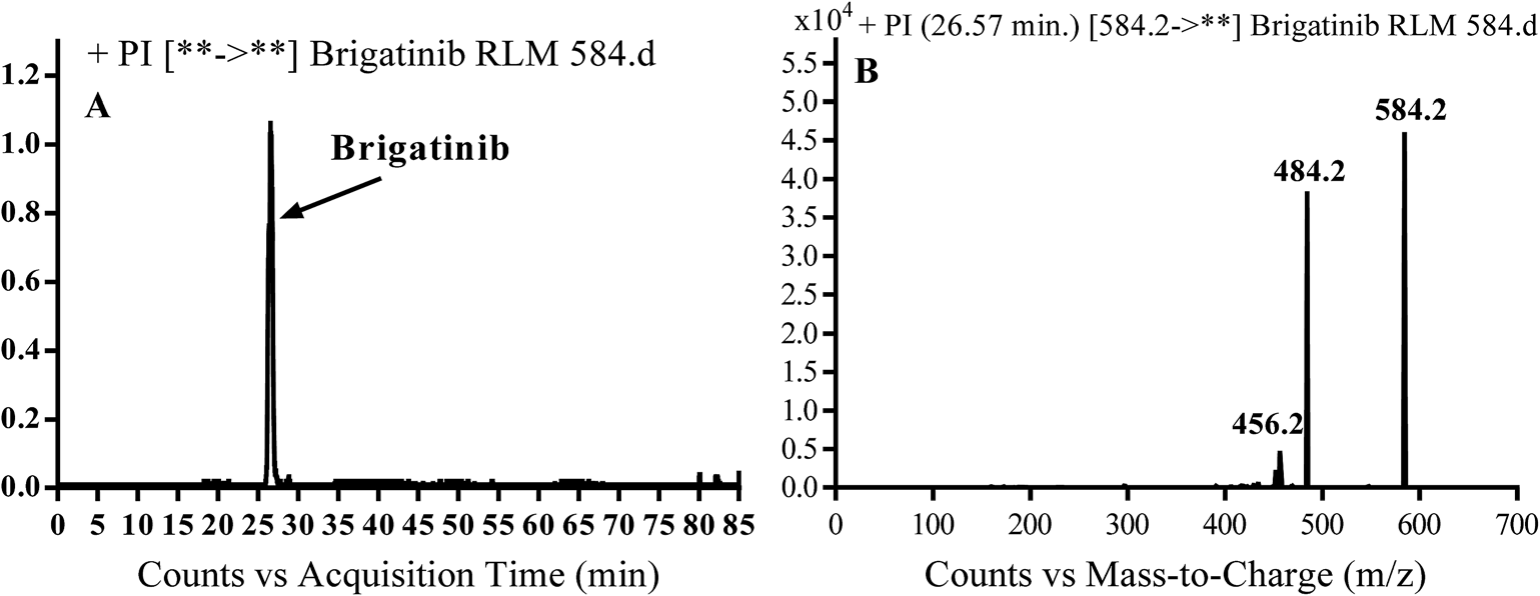
PI chromatogram of MIP at *m*/*z* 584 showing BGB peak at 26.6 min (A), PI mass spectrum of BGB (B).

**Scheme 1:**
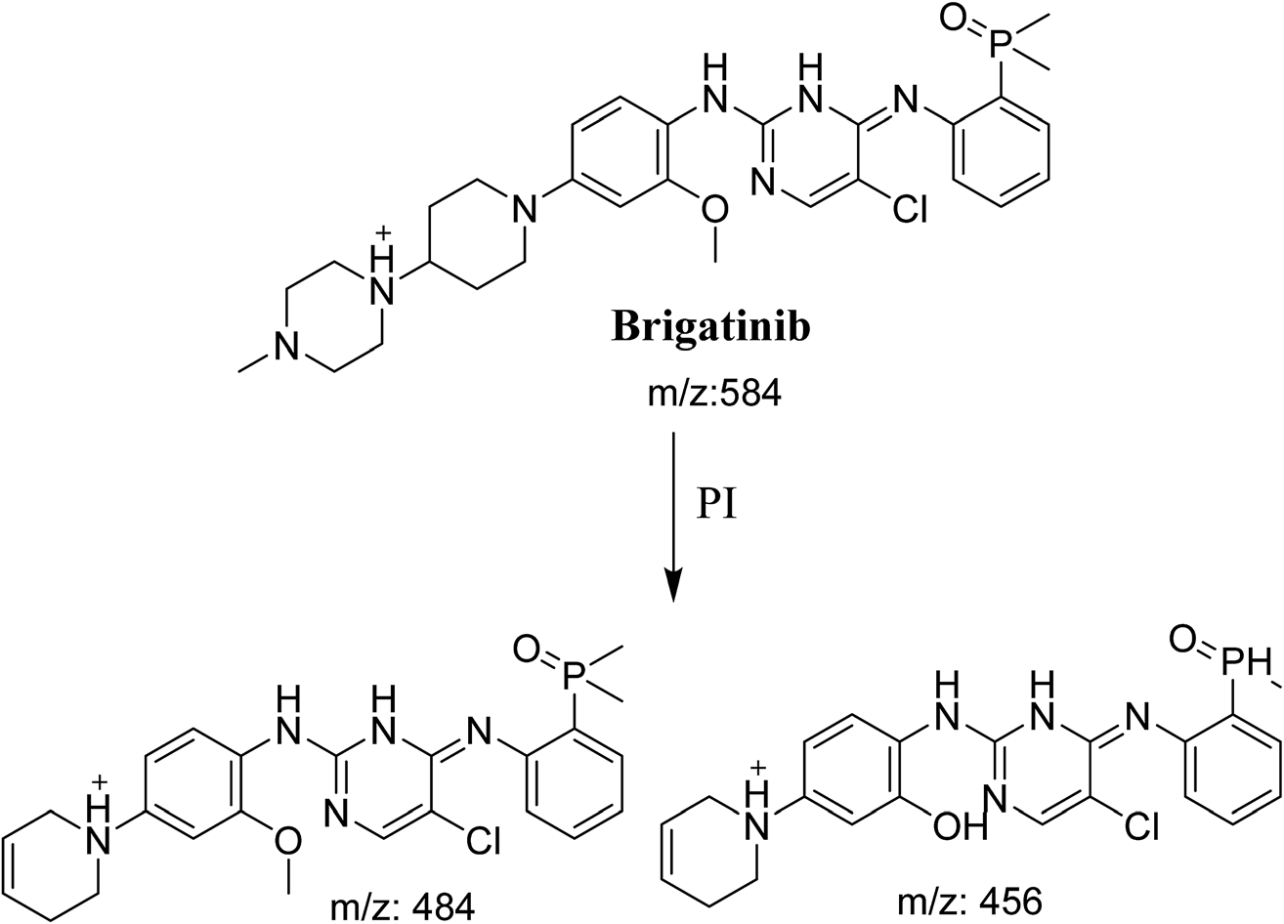
Fragmentation pattern of BGB.

BGB molecular ion peak (MIP) appears as [M + H]^+^ (*m*/*z* 584) at 26.6 min in TIC chromatogram (Fig. 2A). Fragmentation of parent ion at *m*/*z* 584 gave two PI at *m*/*z* 484 and at *m*/*z* 456 (Fig. 2B). The PI at *m*/*z* 484 represents the loss of *N*-methyl piperazine group by single bond cleavage (Scheme 1) as reported in the literature.^33^

### 3.2. Identification of *in vitro* phase I BGB metabolites

Three *in vitro* phase I metabolic pathways generated four metabolites through: *N*-dealkylation, α hydroxylation and α oxidation (Table 2). Metabolites were not observed in control incubations (that does not contains either BGB or RLMs).

**Table 2:** Phase I metabolites of BGB

	MS scan	PIs	Rt. (min)	Metabolic reaction
BGB	584	484, 456	26.6	

**Phase I metabolites**				
BGB600	600	500, 482, 203	32.9	α-Hydroxylation at piperidine ring
BGB570	570	484	29.5	*N*-Demethylation
BGB486	486	458, 394	30.9	*N*-Dealkylation and loss of *N*-methyl piperazine ring
BGB598	598	482, 182	27.3	α-Oxidation at piperidine ring

#### 3.2.1. Identification of BGB570 phase I metabolite of BGB

**Fig. 3:**
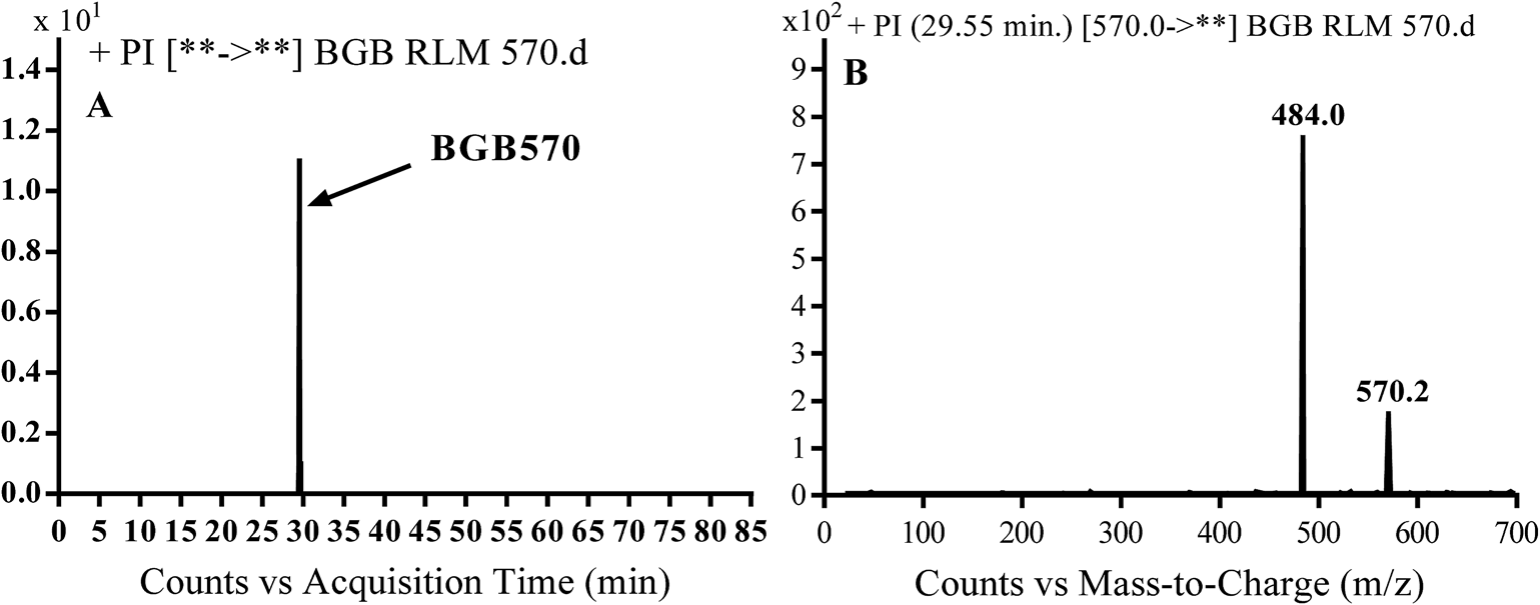
PI chromatogram of MIP at *m*/*z* 570 showing BGB570 peak at 29.5 min (A), PI spectrum of BGB570 (B).

**Scheme 2:**
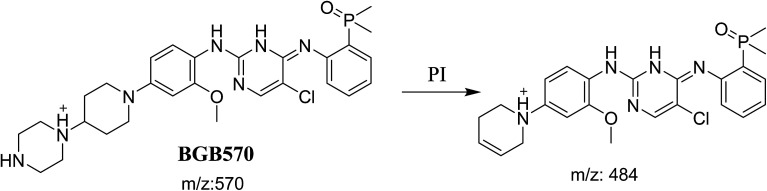
Fragmentation pattern of BGB570.

BGB570 MIP appears as [M + H]^+^ (*m*/*z* 570) at 29.5 min in PI chromatogram (Fig. 3A). Fragmentation of BGB570 gave one PI at *m*/*z* 484 (Fig. 3B) which represents loss of piperazine ring. PI at *m*/*z* 484 proposed that metabolic change occurred in the piperazine ring. The PI at *m*/*z* 484 revealed that *N*-demethylation of *N*-methyl piperazine ring of BGB570 (Scheme 2).

#### 3.2.2. Identification of BGB600 phase I metabolite of BGB

**Fig. 4:**
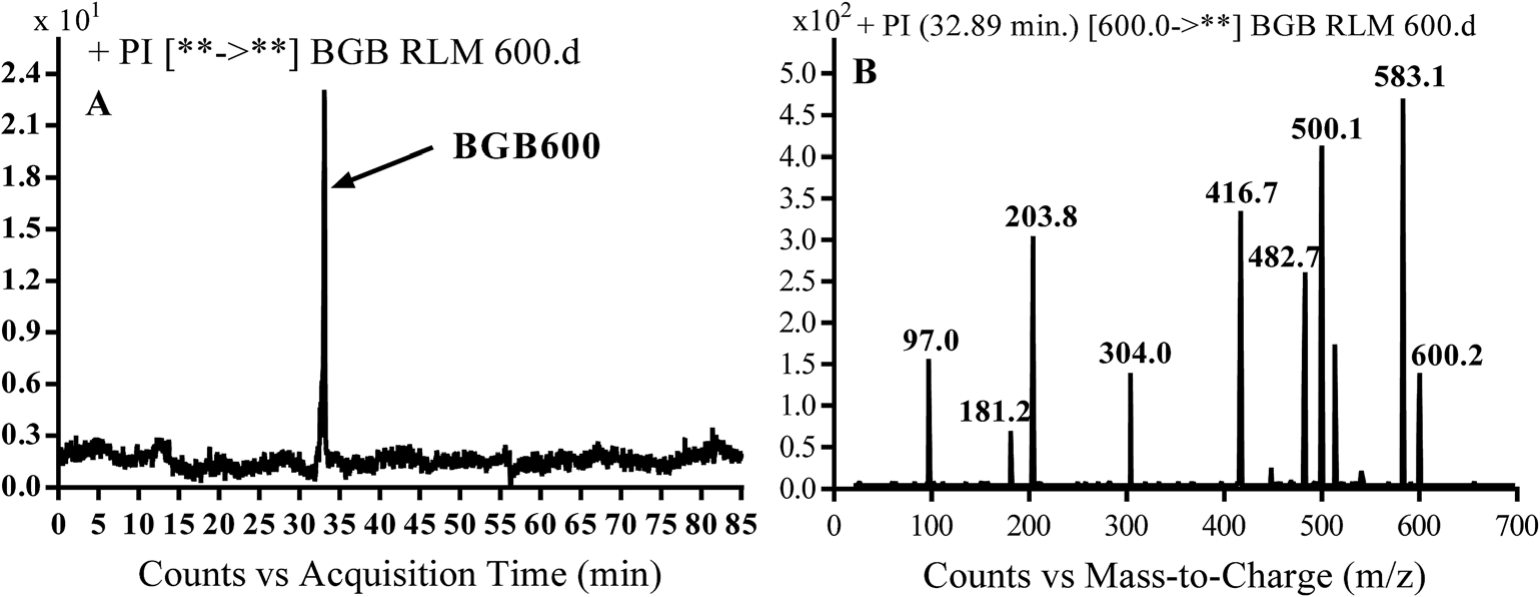
PI chromatogram of MIP at *m*/*z* 600 showing BGB600 peak at 32.9 min (A), PI spectrum of BGB600 (B).

**Scheme 3:**
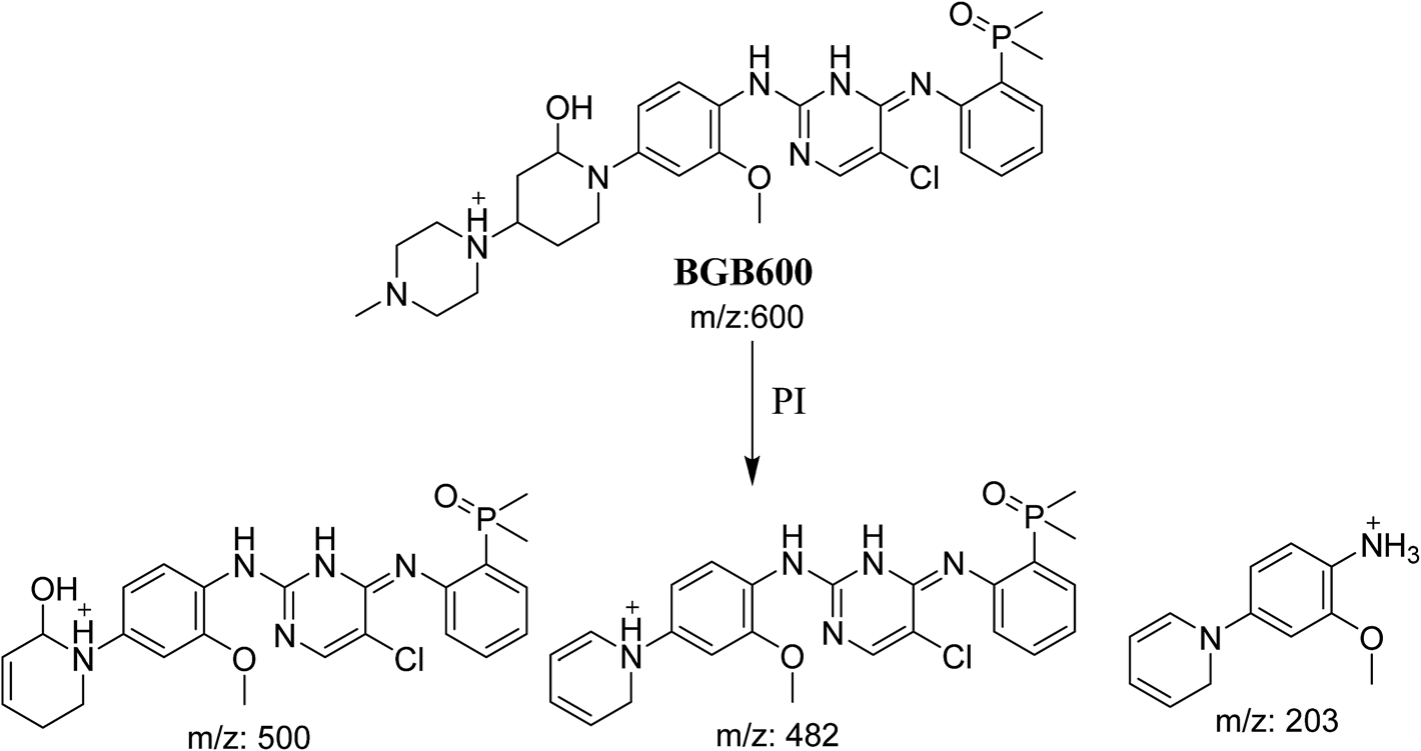
Fragmentation pattern of BGB600.

BGB600 MIP appears as [M + H]^+^ (*m*/*z* 600) at 32.4 min in PI chromatogram (Fig. 4A). Fragmentation of BGB600 at *m*/*z* 600 gave PI at *m*/*z* 500, *m*/*z* 482 and *m*/*z* 203 (Fig. 4B). PI at *m*/*z* 500 proposed that metabolic change was occurred in the piperazine ring. Hydroxylation was proposed to occur in the α position of piperidine nitrogen atom (Scheme 3).

#### 3.2.3. Identification of BGB486 phase I metabolite of BGB

**Fig. 5:**
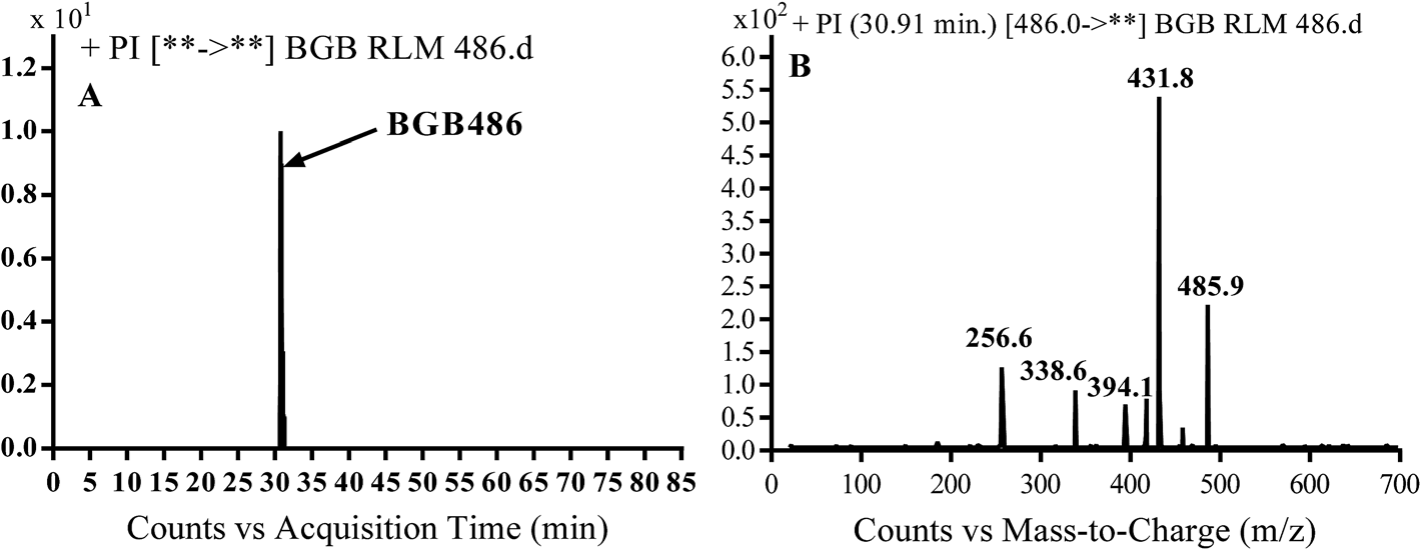
PI chromatogram of MIP at *m*/*z* 486 showing BGB486 peak at 30.9 min (A), PI mass spectrum of BGB486 (B).

**Scheme 4:**
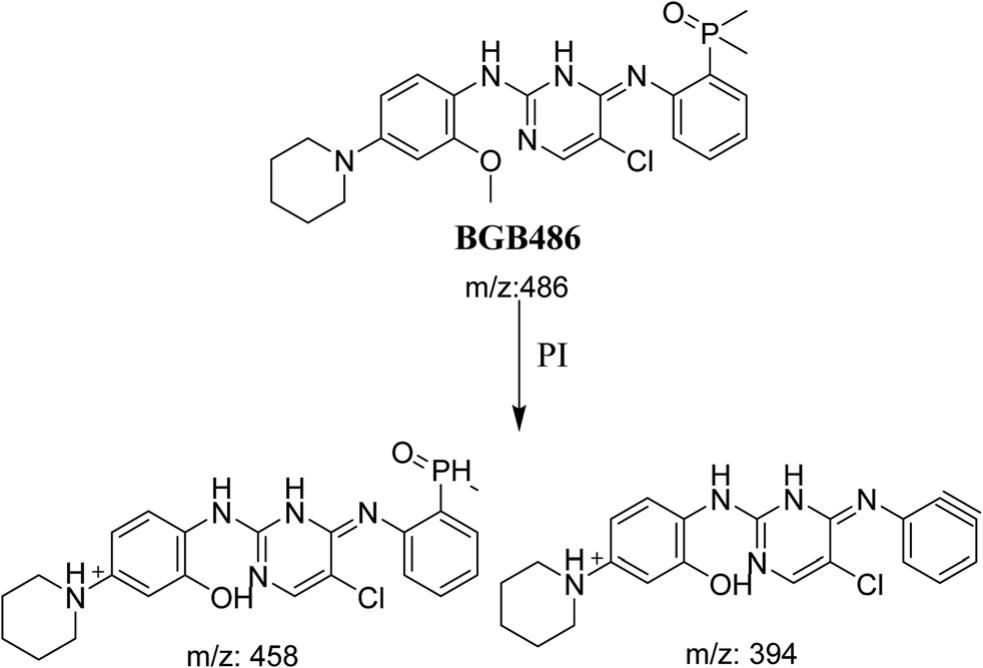
Fragmentation pattern of BGB486.

BGB486 MIP appears as [M + H]^+^ (*m*/*z* 486) at 30.9 min in PI chromatogram (Fig. 5A). Fragmentation of parent ion at *m*/*z* 486 gave PI at *m*/*z* 458 and *m*/*z* 394 (Fig. 5B). PI at 458 proposed that *N*-dealkylation and loss of *N*-methyl piperazine ring (Scheme 4).

#### 3.2.4. Identification of BGB598 phase I metabolite of BGB

**Fig. 6:**
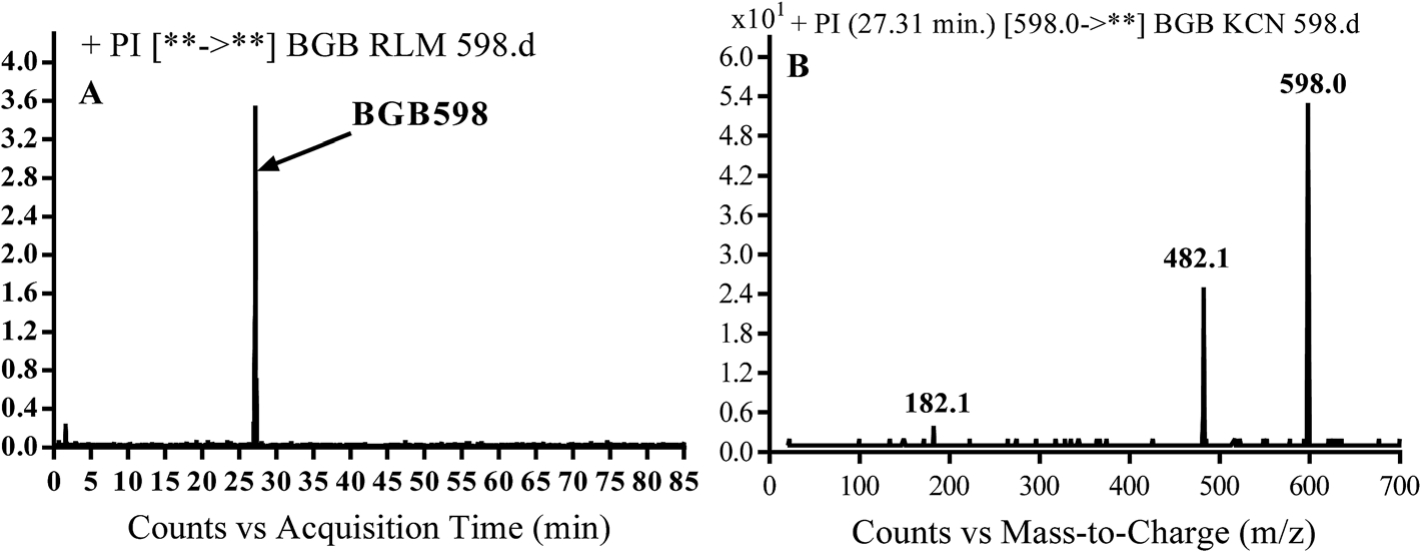
PI chromatogram of MIP at *m*/*z* 486 showing BGB598 peak at 27.3 min (A), PI mass spectrum of BGB598 (B).

**Scheme 5:**
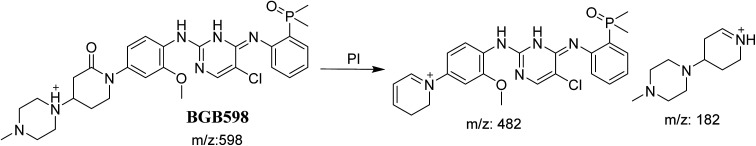
Fragmentation pattern of BGB598.

BGB589 MIP appears as [M + H]^+^ (*m*/*z* 598) at 27.3 min in PI chromatogram (Fig. 6B). Fragmentation of parent ion at *m*/*z* 598 gave PI at *m*/*z* 482 and *m*/*z* 182 (Fig. 6B). PI at *m*/*z* 482 proposed the α oxidation of piperidine group that matched with the other PI at *m*/*z* 182 (Scheme 5).

### 3.3. Reactive metabolites

Three cyano adducts were detected in the case of incubation of BGB with RLMs in the presence of 1.0 mM KCN (Table 3).

**Table 3:** Cyano adducts of BGB

Reactive metabolites				
	MS scan	Most abundant fragment ions	Rt. (min)	Metabolic reaction
BGB609	609	482	31.4	Cyano addition
BGB623	623	596, 482	42.9	Cyano addition and α oxidation at *N*-methyl piperazine ring
BGB527	527	482, 416	43.9	Cyano addition, α hydroxylation at piperidine ring and loss of *N*-methyl piperazine group

#### 3.3.1. Identification of BGB609 cyano adduct of BGB

**Fig. 7:**
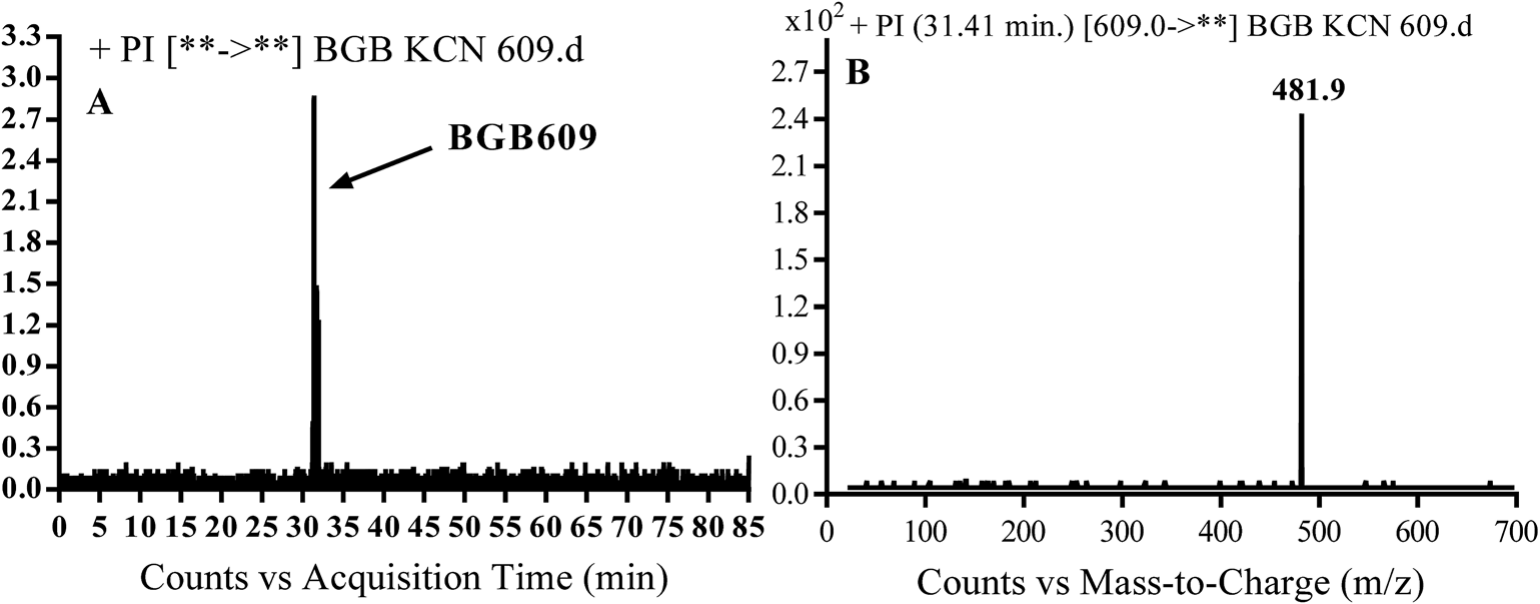
PI chromatogram of MIP at *m*/*z* 609 showing BGB609 peak at 31.4 min (A), PI mass spectrum of BGB609 (B).

**Scheme 6:**
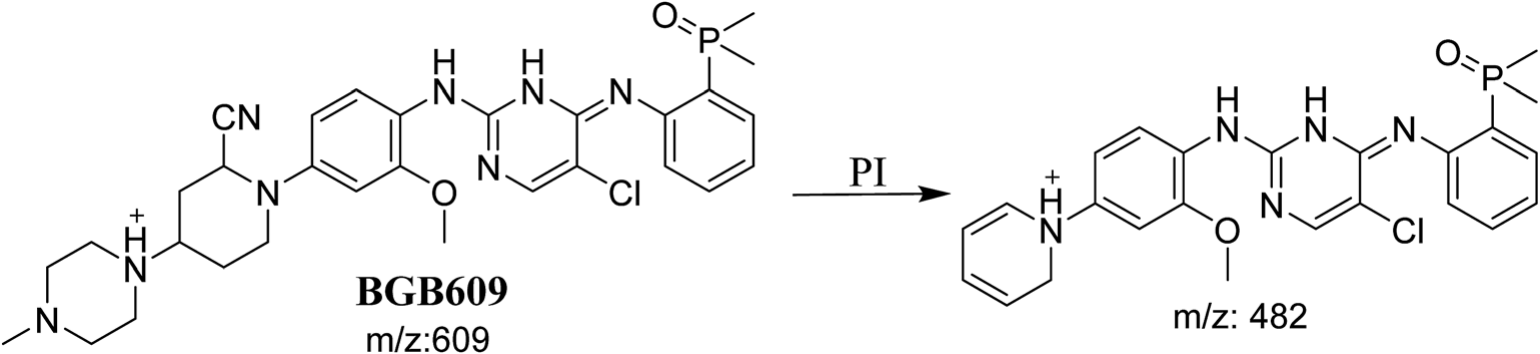
Fragmentation pattern of BGB609.

BGB609 MIP appears as [M + H]^+^ (*m*/*z* 609) at 31.4 min in PI chromatogram (Fig. 7A). Fragmentation of parent ion at *m*/*z* 609 gave PI at *m*/*z* 482 (Fig. 7B). The PI at *m*/*z* 482 represented an immediate elimination of one molecule of HCN in addition to loss of *N*-methyl piperazine ring. The PI at *m*/*z* 482 proposed the addition of cyanide ion at the activated α carbon of the nitrogen atom of piperidine ring (Scheme 6).

#### 3.3.2. Identification of BGB623 cyano adduct of BGB

**Fig. 8:**
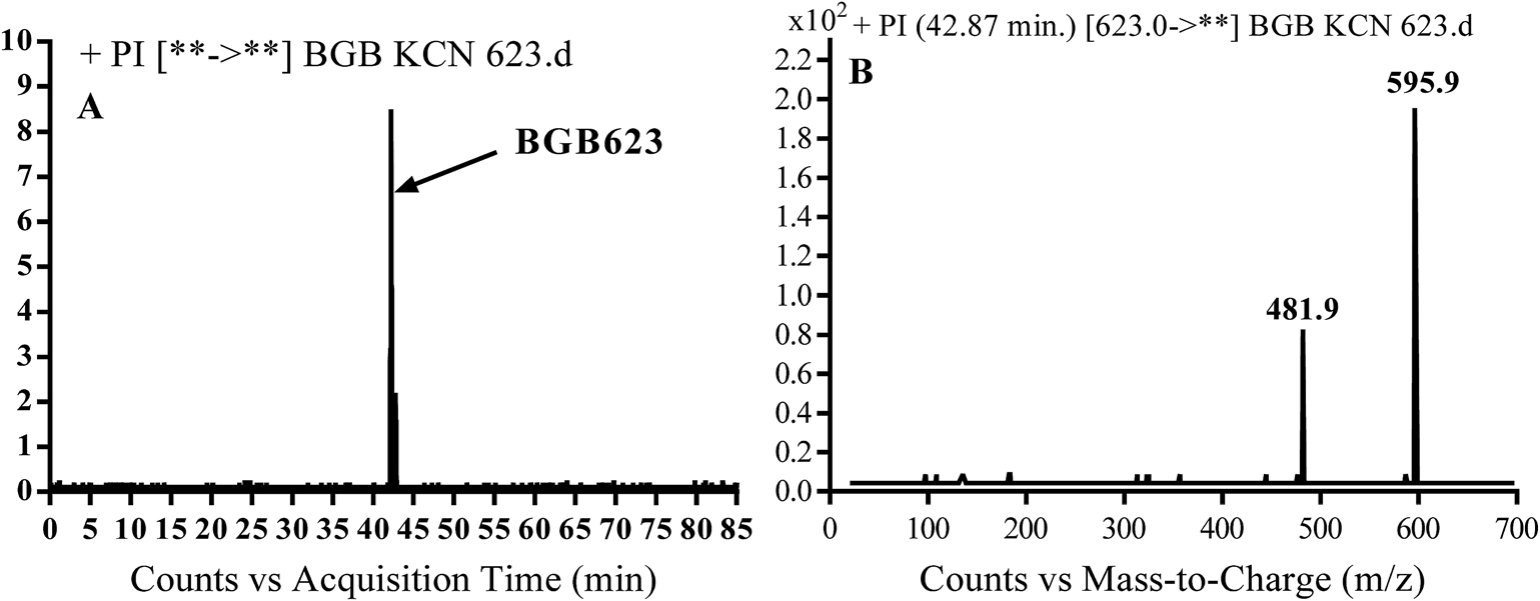
PI chromatogram of MIP at *m*/*z* 623 showing BGB623 peak at 42.8 min (A), PI mass spectrum of BGB623 (B).

**Scheme 7:**
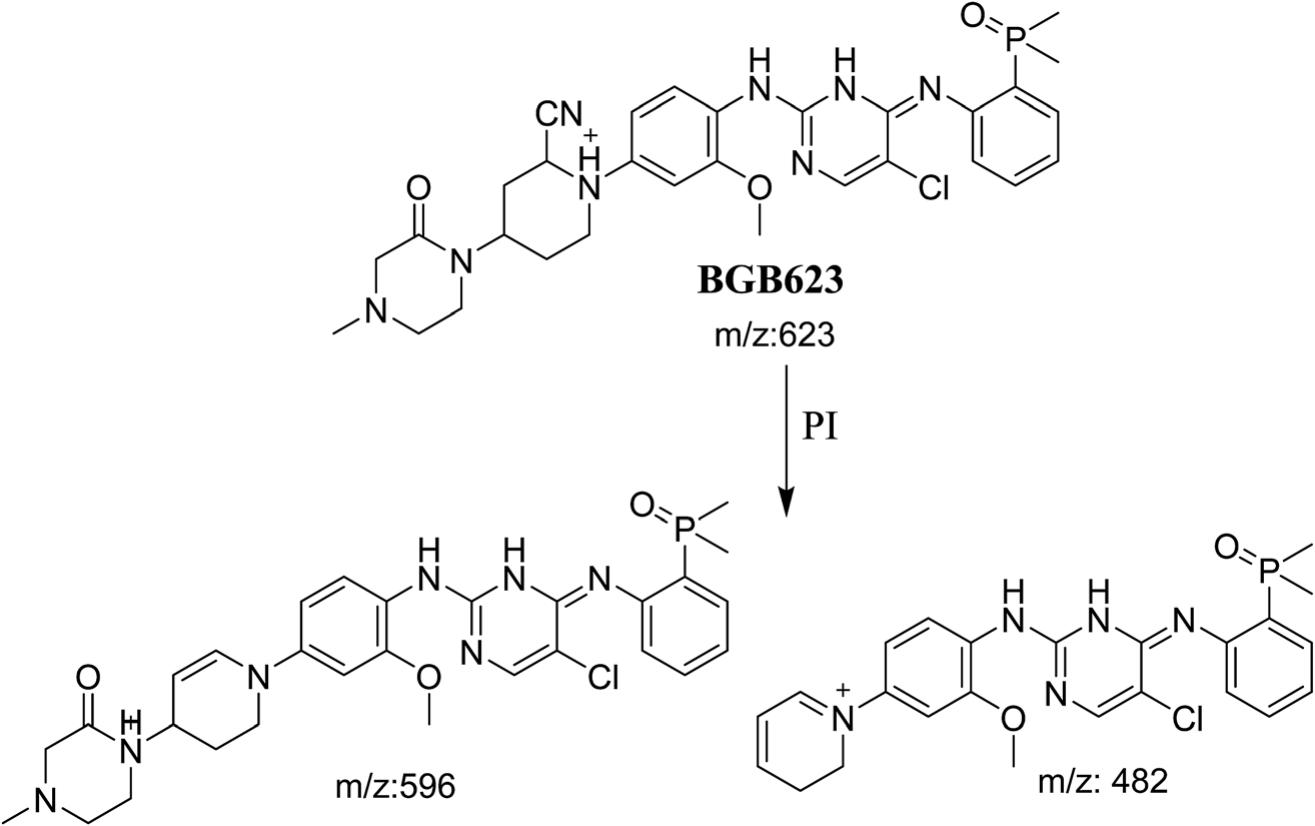
Fragmentation pattern of BGB623.

BGB623 MIP appears as [M + H]^+^ (*m*/*z* 623) at 42.8 min in PI chromatogram (Fig. 8A). Fragmentation of parent ion at *m*/*z* 623 gave PI at *m*/*z* 596 and *m*/*z* 482 (Fig. 8B). The PI at *m*/*z* 596 represented an immediate loss of a molecule of HCN. The PI at *m*/*z* 482 proposed the addition of cyanide ion at the activated α carbon of piperidine ring. The metabolic reaction of BGB623 proposed to be α oxidation of *N*-methyl piperazine ring and addition of cyanide ion at α position in piperidine ring (Scheme 7).

#### 3.3.3. Identification of BGB527 cyano adduct of BGB

**Fig. 9:**
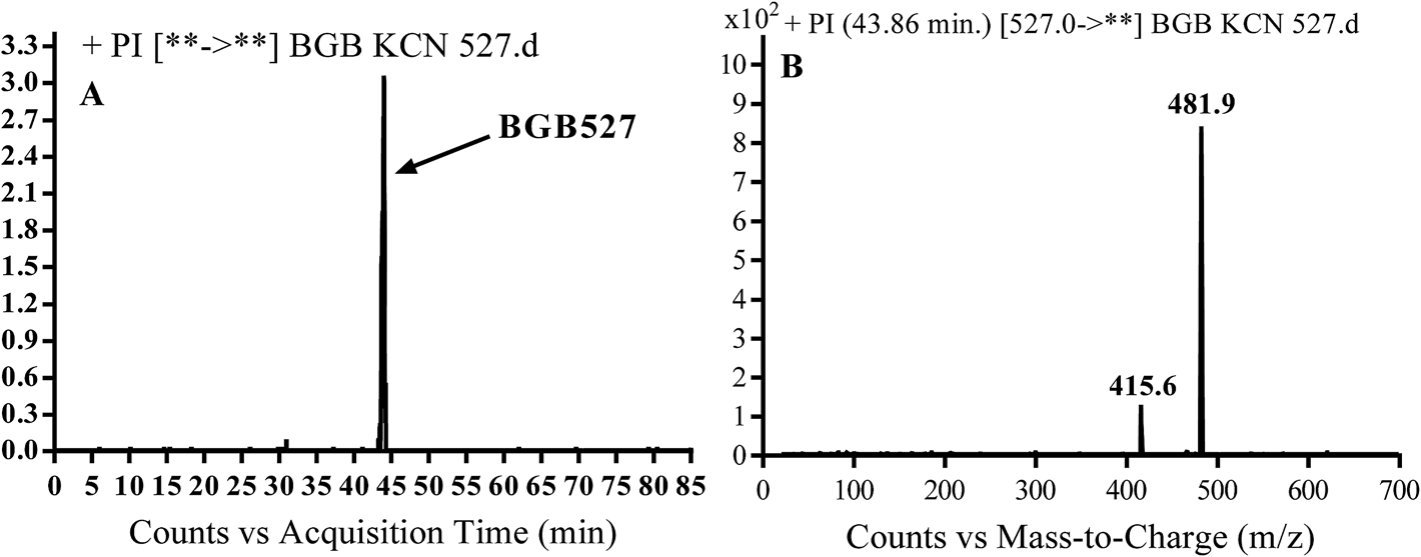
PI chromatogram of MIP at *m*/*z* 527 showing BGB527 peak at 43.9 min (A), PI mass spectrum of BGB527 (B).

**Scheme 8:**
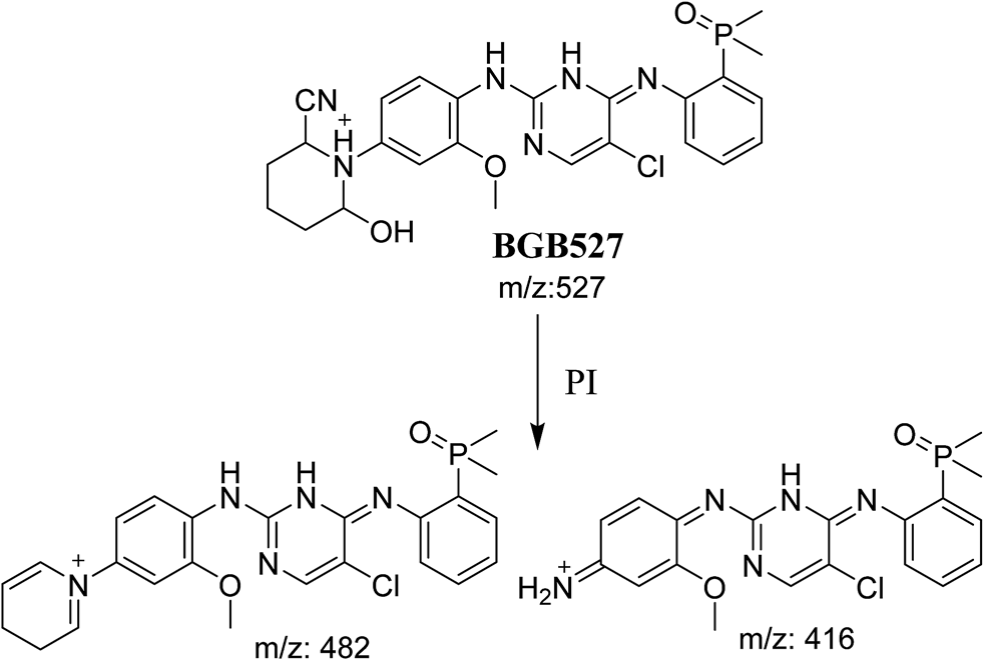
Fragmentation pattern of BGB527.

BGB527 MIP appears as [M + H]^+^ (*m*/*z* 527) at 43.9 min in PI chromatogram (Fig. 9A). Fragmentation of parent ion at *m*/*z* 527 gave PI at *m*/*z* 482 and *m*/*z* 416 (Fig. 9B). The PI at *m*/*z* 482 proposed that the addition of cyanide ion at the activated α carbon of the N atom of piperidine ring. The metabolic reaction of BGB527 proposed to be α hydroxylation and α addition of cyanide ion at piperidine ring, and loss of *N*-methyl piperazine ring through *N*-dealkylation metabolic reaction (Scheme 8).

### 3.4. Mechanism of bioactivation of BGB

**Scheme 9:**
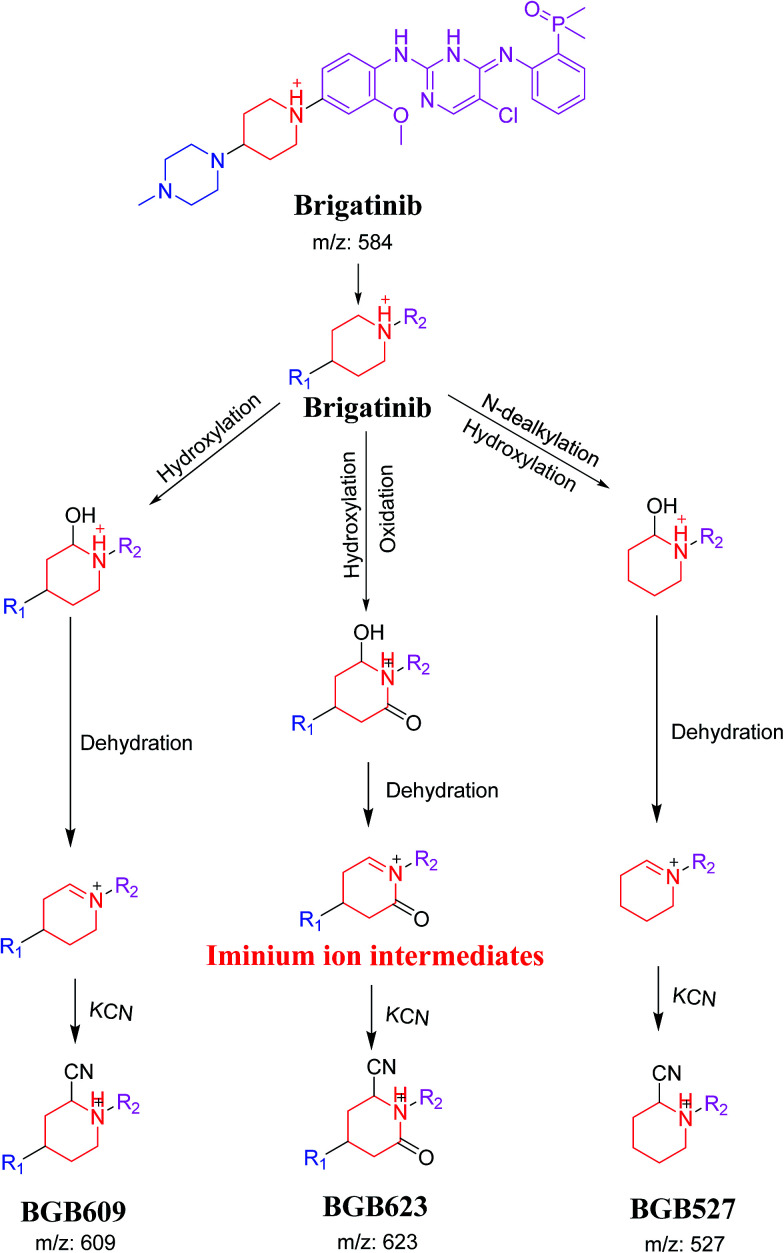
Proposed mechanism of bioactivation of BGB into reactive intermediates and trapping strategy.

The formation of BGB527, BGB609 and BGB623 cyanide adduct indicated that the formation of iminium intermediates in the piperidine ring. α Hydroxylation of piperidine ring in BGB then dehydration resulted in formation of iminium ions (BGB527 and BGB609) or iminium carbonyl (BGB623) that are not stable but can be trapped by cyanide forming stable adduct (Scheme 9). The mechanism of BGB bioactivation is previously described with other similar cyclic tertiary amine containing drugs.^34–36^

## 4. Conclusions

**Fig. 10:**
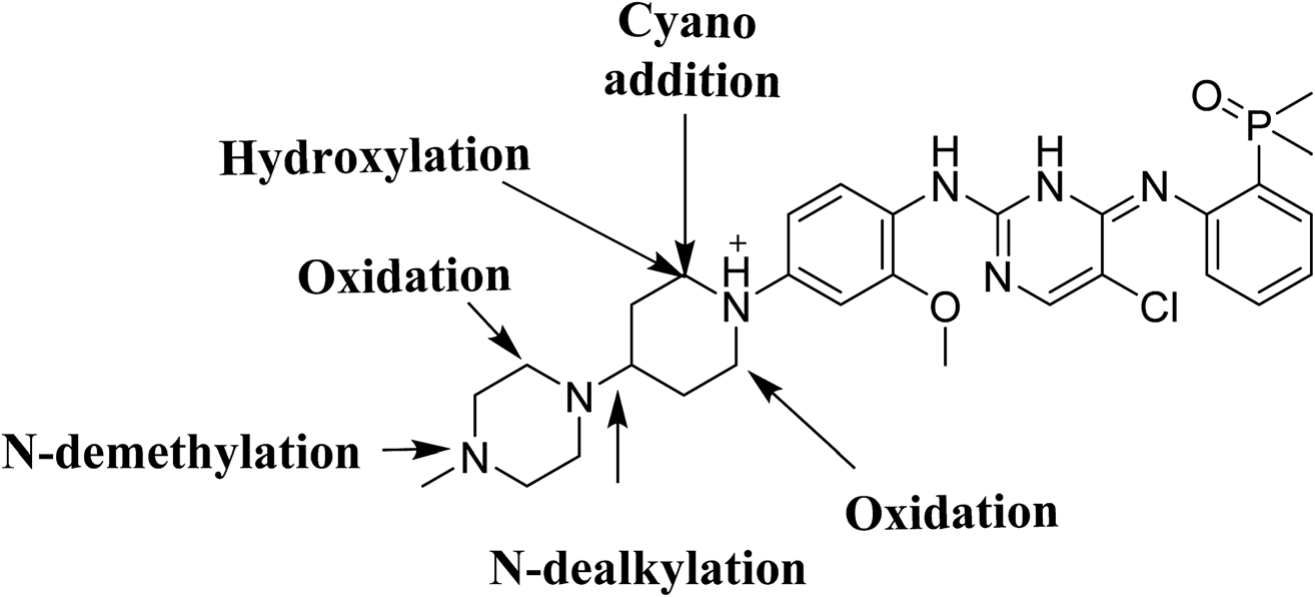
Chemical structure of BGB showing places of phase I metabolic reaction and bioactivation pathways.

Four phase I BGB metabolites and three cyano adducts for BGB were detected using LC-MS/MS (Fig. 10). Piperidine ring was found to be responsible of BGB bioactivation. This study illuminates the way for further work about BGB side effects. Isosteric replacements at both 2 α carbons of piperidine group can retain activity with reduce side effects by blocking bioactivation pathways.

## Conflicts of interest

The authors declare no conflict of interest.

## Supplementary Material
